# Supplementary material for: Gender differences influence over insomnia in Korean population: A cross-sectional study
Source: PLoS One. 2020 Jan 9;15(1):e0227190. doi: 10.1371/journal.pone.0227190 (PMC6952093; doi:10.1371/journal.pone.0227190)
Supplement: S1 Table — In Model 1, adjustment was conducted for sociodemographic variables (age, sex, size of residential area and educational level) and short sleep time. Model 2 incorporated anxiety (GAS score) with Model 1. Model 3 included depression (PHQ-9 score ≥ 10) with Model 1. The final model, Model 4, incorporated poor sleep quality (PSQI score ≥ 6), anxiety and depression with Model 1. Subject with missing data was excluded from the analysis. p was calculated by the univariable / multiple logistic regression analysis. Abbreviations: OR = odds ratio, CI = confidence interval. (DOCX) [file pone.0227190.s001.docx]

**Supplementary table 1.** Univariable and multivariable regression analysis for

difficulty initiating sleep (DIS)

|  | **Univariable ORs** | |  | **Multivariable analysis ORs** | | | | | | |
| --- | --- | --- | --- | --- | --- | --- | --- | --- | --- | --- |
|  |  | | Model 1 | | Model 2 | | Model 3 | | Model4 | |
|  | OR (95%Ci) | p-value | OR (95%Ci) | p-value | OR (95%Ci) | p-value | OR (95%Ci) | p-value | OR (95%Ci) | p-value |
| **Sex (Women)** | 1.689  (1.240-2.302) | 0.001 | 1.628  (1.183-2.240) | 0.003 | 1.523  (1.089-2.131) | 0.014 | 1.472  (1.045-2.074) | 0.027 | 1.425  (1.002-2.027) | 0.049 |
| **Age**  **(40 years or older)** | 1.507  (1.097-2.071) | 0.011 | 1.105  (0.777-1.571) | 0.580 | 1.144  (0.792-1.652) | 0.474 | 1.300  (0.888-1.903) | 0.177 | 1.266  (0.858-1.868) | 0.235 |
| **Size of residential area**  **(Large city)** | 1.017  (0.752-1.375) | 0.912 | 1.022  (0.750-1.393) | 0.891 | 0.981  (0.707-1.360) | 0.907 | 0.976  (0.698-1.365) | 0.887 | 0.947  (0.671-1.337) | 0.757 |
| **Education**  **(Middle school**  **or less)** | 1.918  (1.337-2.751) | <0.001 | 1.565  (1.047-2.340) | 0.029 | 1.430  (0.937-2.182) | 0.097 | 1.565  (1.016-2.410) | 0.042 | 1.468  (0.940-2.293) | 0.092 |
| **Sleep duration**  **(6 hours**  **or shorter)** | 3.868  (2.826-5.293) | <0.001 | 3.755  (2.722-5.178) | <0.001 | 3.267  (2.317-4.606) | <0.001 | 3.301  (2.320-4.698) | <0.001 | 3.088  (2.148-4.441) | <0.001 |
| **Anxiety** | 9.975  (7.185-13.849) | <0.001 |  |  | 8.875  (6.317-12.470) | <0.001 |  |  | 4.983  (3.384-7.339) | <0.001 |
| **Depression** | 20.668  (13.764-31.036) | <0.001 |  |  |  |  | 19.412  (12.612-29.877) | <0.001 | 9.614  (5.950-15.532) | <0.001 |

In Model 1, adjustment was conducted for sociodemographic variables (age, sex, size of residential area and educational level) and short sleep time. Model 2 incorporated anxiety (GAS score) with Model 1. Model 3 included depression (PHQ-9 score ≥ 10) with Model 1. The final model, Model 4, incorporated poor sleep quality (PSQI score ≥ 6), anxiety and depression with Model 1. Subject with missing data was excluded from the analysis.

*p* was calculated by the univariable / multiple logistic regression analysis. *Abbreviations*: OR = odds ratio, CI = confidence interval.
