# Supplementary material for: Gender differences influence over insomnia in Korean population: A cross-sectional study
Source: PLoS One. 2020 Jan 9;15(1):e0227190. doi: 10.1371/journal.pone.0227190 (PMC6952093; doi:10.1371/journal.pone.0227190)
Supplement: S2 Table — In Model 1, adjustment was conducted for sociodemographic variables (age, sex, size of residential area and educational level) and short sleep time. Model 2 incorporated anxiety (GAS score) with Model 1. Model 3 included depression (PHQ-9 score ≥ 10) with Model 1. The final model, Model 4, incorporated poor sleep quality (PSQI score ≥ 6), anxiety and depression with Model 1. Subject with missing data was excluded from the analysis. p was calculated by the univariable / multiple logistic regression analysis. Abbreviations: OR = odds ratio, CI = confidence interval. (DOCX) [file pone.0227190.s002.docx]

**Supplementary table 2.** Univariable and multivariable regression analysis for

difficulty maintaining sleep (DMS)

|  | **Univariable ORs** | |  | **Multivariable analysis ORs** | | | | | | |
| --- | --- | --- | --- | --- | --- | --- | --- | --- | --- | --- |
|  |  | | Model 1 | | Model 2 | | Model 3 | | Model4 | |
|  | OR (95%Ci) | p-value | OR (95%Ci) | p-value | OR (95%Ci) | p-value | OR (95%Ci) | p-value | OR (95%Ci) | p-value |
| **Sex (Women)** | 1.994  (1.446-2.751) | <0.001 | 1.965  (1.410-2.736) | <0.001 | 1.866  (1.318-2.643) | <0.001 | 1.828  (1.284-2.603) | 0.001 | 1.787  (1.242-2.569) | 0.002 |
| **Age**  **(40 years or older)** | 1.452  (1.052-2.003) | 0.023 | 1.112  (0.777-1.592) | 0.562 | 1.158  (0.796-1.683) | 0.444 | 1.310  (0.891-1.928) | 0.170 | 1.281  (0.863-1.899) | 0.219 |
| **Size of residential area**  **(Large city)** | 0.928  (0.682-1.263) | 0.634 | 0.936  (0.682-1.284) | 0.681 | 0.884  (0.633-1.235) | 0.470 | 0.878  (0.624-1.235) | 0.455 | 0.843  (0.593-1.198) | 0.340 |
| **Education**  **(Middle school**  **or less)** | 1.907  (1.319-2.757) | 0.001 | 1.513  (1.005-2.279) | 0.047 | 1.386  (0.902-2.129) | 0.136 | 1.507  (0.974-2.331) | 0.065 | 1.404  (0.894-2.205) | 0.140 |
| **Sleep duration**  **(6 hours**  **or shorter)** | 3.269  (2.367-4.514) | <0.001 | 3.214  (2.305-4.482) | <0.001 | 2.730  (1.913-3.896) | <0.001 | 2.712  (1.885-3.902) | <0.001 | 2.507  (1.722-3.650) | <0.001 |
| **Anxiety** | 10.149  (7.278-14.154) | <0.001 |  |  | 9.158  (6.494-12.915) | <0.001 |  |  | 5.247  (3.554-7.746) | <0.001 |
| **Depression** | 19.294  (12.830-29.015) | <0.001 |  |  |  |  | 17.947  (11.679-27.578) | <0.001 | 8.575  (5.301-13.871) | <0.001 |

In Model 1, adjustment was conducted for sociodemographic variables (age, sex, size of residential area and educational level) and short sleep time. Model 2 incorporated anxiety (GAS score) with Model 1. Model 3 included depression (PHQ-9 score ≥ 10) with Model 1. The final model, Model 4, incorporated poor sleep quality (PSQI score ≥ 6), anxiety and depression with Model 1. Subject with missing data was excluded from the analysis.

*p* was calculated by the univariable / multiple logistic regression analysis. *Abbreviations*: OR = odds ratio, CI = confidence interval.
