# Supplementary material for: Gender differences influence over insomnia in Korean population: A cross-sectional study
Source: PLoS One. 2020 Jan 9;15(1):e0227190. doi: 10.1371/journal.pone.0227190 (PMC6952093; doi:10.1371/journal.pone.0227190)
Supplement: S3 Table — In Model 1, adjustment was conducted for sociodemographic variables (age, sex, size of residential area and educational level) and short sleep time. Model 2 incorporated anxiety (GAS score) with Model 1. Model 3 included depression (PHQ-9 score ≥ 10) with Model 1. The final model, Model 4, incorporated poor sleep quality (PSQI score ≥ 6), anxiety and depression with Model 1. Subject with missing data was excluded from the analysis. p was calculated by the univariable / multiple logistic regression analysis. Abbreviations: OR = odds ratio, CI = confidence interval. (DOCX) [file pone.0227190.s003.docx]

**Supplementary table 3.** Univariable and multivariable regression analysis for

early morning awakening (EMA)

|  | **Univariable ORs** | |  | **Multivariable analysis ORs** | | | | | | |
| --- | --- | --- | --- | --- | --- | --- | --- | --- | --- | --- |
|  |  | | Model 1 | | Model 2 | | Model 3 | | Model4 | |
|  | OR (95%Ci) | p-value | OR (95%Ci) | p-value | OR (95%Ci) | p-value | OR (95%Ci) | p-value | OR (95%Ci) | p-value |
| **Sex (Women)** | 1.633  (1.194-2.234) | 0.002 | 1.581  (1.139-2.194) | 0.006 | 1.509  (1.071-2.128) | 0.019 | 1.461  (1.037-2.059) | 0.030 | 1.435  (1.009-2.043) | 0.045 |
| **Age**  **(40 years or older)** | 2.124  (1.507-2.994) | <0.001 | 1.515  (1.037-2.214) | 0.032 | 1.606  (1.084-2.379) | 0.018 | 1.771  (1.187-2.641) | 0.005 | 1.755  (1.168-2.637) | 0.007 |
| **Size of residential area**  **(Large city)** | 0.873  (0.642-1.189) | 0.390 | 0.892  (0.647-1.229) | 0.485 | 0.845  (0.603-1.184) | 0.328 | 0.844  (0.602-1.183) | 0.325 | 0.813  (0.574-1.151) | 0.243 |
| **Education**  **(Middle school**  **or less)** | 2.394  (1.684-3.403) | <0.001 | 1.726  (1.167-2.554) | 0.006 | 1.615  (1.072-2.432) | 0.022 | 1.730  (1.149-2.605) | 0.009 | 1.643  (1.078-2.505) | 0.021 |
| **Sleep duration**  **(6 hours**  **or shorter)** | 4.683  (3.416-6.420) | <0.001 | 4.413  (3.191-6.104) | <0.001 | 3.915  (2.778-5.519) | <0.001 | 3.922  (2.782-5.530) | <0.001 | 3.719  (2.612-5.296) | <0.001 |
| **Anxiety** | 8.949  (6.415-12.484) | <0.001 |  |  | 8.090  (5.691-11.499) | <0.001 |  |  | 5.105  (3.446-7.563) | <0.001 |
| **Depression** | 12.937  (8.579-19.509) | <0.001 |  |  |  |  | 12.508  (7.984-19.596) | <0.001 | 5.684  (3.430-9.420) | <0.001 |

In Model 1, adjustment was conducted for sociodemographic variables (age, sex, size of residential area and educational level) and short sleep time. Model 2 incorporated anxiety (GAS score) with Model 1. Model 3 included depression (PHQ-9 score ≥ 10) with Model 1. The final model, Model 4, incorporated poor sleep quality (PSQI score ≥ 6), anxiety and depression with Model 1. Subject with missing data was excluded from the analysis.

*p* was calculated by the univariable / multiple logistic regression analysis. *Abbreviations*: OR = odds ratio, CI = confidence interval.
