# Supplementary material for: Gender differences influence over insomnia in Korean population: A cross-sectional study
Source: PLoS One. 2020 Jan 9;15(1):e0227190. doi: 10.1371/journal.pone.0227190 (PMC6952093; doi:10.1371/journal.pone.0227190)
Supplement: S4 Table — Subject with missing data was excluded from the analysis. p was calculated by the univariable / multiple logistic regression analysis. Abbreviations: OR = odds ratio, CI = confidence interval, IS = Insomnia Symptoms, DIS = Difficulty in Initiating Sleep, DMS = Difficulty in Maintaining Sleep, EMA = Early Morning Awakening. (DOCX) [file pone.0227190.s004.docx]

**Supplementary table 4.** Univariable and multivariable regression analysis

for insomnia symptoms and insomnia subtypes in women

|  | **Multivariable analysis ORs** | | | | | | | |
| --- | --- | --- | --- | --- | --- | --- | --- | --- |
|  | IS | | DIS | | DMS | | EMA | |
|  | OR (95%Ci) | p-value | OR (95%Ci) | p-value | OR (95%Ci) | p-value | OR (95%Ci) | p-value |
| **Age**  **(40 years or older)** | 1.195  (0.764-1.869) | 0.435 | 1.191  (0.684-2.074) | 0.536 | 1.165  (0.683-1.989) | 0.576 | 1.620  (0.922-2.846) | 0.093 |
| **Size of residential area**  **(Large city)** | 0.811  (0.559-1.176) | 0.269 | 0.735  (0.465-1.163) | 0.188 | 0.762  (0.486-1.196) | 0.237 | 0.808  (0.515-1.267) | 0.353 |
| **Education**  **(Middle school**  **or less)** | 1.473  (0.877-2.473) | 0.143 | 1.532  (0.826-2.840) | 0.176 | 1.176  (0.628-2.203) | 0.612 | 1.771  (0.991-3.166) | 0.054 |
| **Sleep duration**  **(6 hours**  **or shorter)** | 2.282  (1.501-3.471) | <0.001 | 3.147  (1.944-5.097) | <0.001 | 2.395  (1.466-3.912) | <0.001 | 3.345  (2.099-5.329) | <0.001 |
| **Anxiety** | 6.024  (3.935-9.222) | <0.001 | 4.685  (2.817-7.792) | <0.001 | 5.187  (3.169-8.488) | <0.001 | 4.267  (2.538-7.174) | <0.001 |
| **Depression** | 6.798  (3.761-12.290) | <0.001 | 8.119  (4.366-15.097) | <0.001 | 7.796  (4.243-14.328) | <0.001 | 4.763  (2.485-9.130) | <0.001 |
| **Monthly income**  **(<2,000 dollar)** | 1.122  (0.668-1.885) | 0.663 | 1.467  (0.807-2.668) | 0.209 | 1.623  (0.896-2.941) | 0.110 | 1.142  (0.633-2.060) | 0.660 |
| **Occupation**  **(unemployed)** | 1.506  (0.795-2.852) | 0.209 | 1.424  (0.636-3.191) | 0.390 | 1.445  (0.666-3.135) | 0.351 | 1.094  (0.445-2.688) | 0.844 |

Subject with missing data was excluded from the analysis.

*p* was calculated by the univariable / multiple logistic regression analysis.

*Abbreviations*: OR = odds ratio, CI = confidence interval, IS = Insomnia Symptoms, DIS = Difficulty in Initiating Sleep, DMS = Difficulty in Maintaining Sleep, EMA = Early Morning Awakening

.
